# Fast analysis of 29 polycyclic aromatic hydrocarbons (PAHs) and nitro-PAHs with ultra-high performance liquid chromatography-atmospheric pressure photoionization-tandem mass spectrometry

**DOI:** 10.1038/srep12992

**Published:** 2015-08-12

**Authors:** Shih-Chun Candice Lung, Chun-Hu Liu

**Affiliations:** 1 Research Center for Environmental Changes, Academia Sinica, Nankang, Taipei, Taiwan; 2 Department of Atmospheric Sciences, National Taiwan University, Taipei, Taiwan

## Abstract

Polycyclic aromatic hydrocarbons (PAHs) and nitro-PAHs are ubiquitous in the environment. Some of them are probable carcinogens and some are source markers. This work presents an ultra-high performance liquid chromatography-atmospheric pressure photoionization-tandem mass spectrometry (UHPLC-APPI-MS/MS) method for simultaneous analysis of 20 PAHs and nine nitro-PAHs. These compounds are separated in 15 minutes in the positive mode and 11 minutes in the negative mode, one half of GC/MS analysis time. Two pairs of precursor/product ions are offered, which is essential for confirmation. This method separates and quantifies benzo[*a*]pyrene (the most toxic PAHs) and non-priority benzo[*e*]pyrene (isomers, little toxicity) to avoid overestimation of toxin levels, demonstrating its importance for health-related researches. With 0.5% 2,4-difluoroanisole in chlorobenzene as the dopant, limits of detection of PAHs except acenaphthylene and those of nitro-PAHs except 2-nitrofluoranthene are below 10 pg and 3 pg, respectively, mostly lower than or comparable to those reported using LC-related systems. The responses were linear over two orders of magnitude with fairly good accuracy and precision. Certified reference materials and real aerosol samples were analyzed to demonstrate its applicability. This fast, sensitive, and reliable method is the first UHPLC-APPI-MS/MS method capable of simultaneously analyzing 29 environmentally and toxicologically important PAHs and nitro-PAHs.

Polycyclic aromatic hydrocarbons (PAHs) are from both natural and anthropogenic sources, typically formed during incomplete combustion^1^; the potential toxicity of PAHs includes carcinogenic, mutagenic, immunologic and reproductive effects^2^,^3^,^4^. In addition, nitrated PAHs (nitro-PAHs) in the air are either formed from their parent PAH compounds by atmospheric reactions or directly emitted from combustion sources such as diesel and gasoline engines^5^,^6^,^7^; certain nitro-PAHs exhibit more toxic potential than their parent PAH compounds^6^,^8^,^9^. PAHs and nitro-PAHs in the air can be transported to regions far away from the sources via atmospheric transportation and deposition processes^1^. Current environmental studies mostly focused on the 16 priority PAHs designated by USEPA^10^,^11^,^12^,^13^; studies on ambient nitro-PAHs are especially limited partially due to analytical difficulty^14^. However, it is crucial to assess environmental levels of toxic non-priority PAHs/nitro-PAHs and source marker PAHs in aerosols to evaluate health risks and apportion different source contributions. Even though increasing research focusing on nitro-PAHs^15^,^16^,^17^,^18^, a single methodology capable of simultaneously analyzing important PAHs and nitro-PAHs is rarely seen. This paper presents a method for simultaneous analysis of 29 important PAHs and nitro-PAHs in aerosols with ultra-high performance liquid chromatography - atmospheric pressure photoionization-tandem mass spectrometry (UHPLC-APPI-MS/MS).

Currently, PAHs and nitro-PAHs in environmental matrix are mostly analyzed by gas chromatography with flame ionization detection (GC-FID) and MS, and high-performance liquid chromatography (HPLC) with UV and fluorescence detectors (FLD)^10^,^11^,^12^,^13^,^14^,^15^,^16^,^17^,^18^. However, GC analysis has proven difficult for those chemicals with molecular weight (m.w.) greater than 300 amu due to their low volatilities^19^. The temperature of injection port is typically set at 350 °C; thus, compounds with m.w. greater than 300 amu is difficult to be vaporized and analyzed by GC-related methods. In fact, the sensitivity of GC analysis for compounds with m.w. greater than 250 amu, such as those PAHs and nitro-PAHs in aerosols, is far from optimal^3^,^20^. On the other hand, HPLC-UV and HPLC-FLD suffer from uncertainty of identification due to possible interference from other compounds^10^,^12^.

The technology of LC/MS can overcome these limitations. Among the three popular ionization sources used in LC/MS, APPI is superior than electrospray ionization (ESI) and atmospheric pressure chemical ionization (APCI) to effectively ionize nonpolar compounds like PAHs^19^,^21^. To date, applying state-of-the-art LC-APPI-MS/MS methods for PAHs and nitro-PAHs analysis are still limited e.g.^11^,^19^,^21^,^22^,^23^,^24^,^25^,^26^,^27^,^28^. Tandem MS has great advantages of providing two pairs of precursor/product ions, which is important for confirmation since there may be other interferences. However, the second pairs of precursor/product ions were only reported in very limited publications 11,22,23,25,26,27. Additionally, the responses of the second product ions reported representing the loss of one proton from the first product ions, may not be intense enough for confirmation in other instruments since the principle of ionization for different LC-MS/MS systems are different. This work searches for other fragments as the second product ions for confirmation using a widely used LC-MS/MS instrument in the analytical laboratories. Three dopants used in APPI were evaluated in terms of limits of detection (LOD), linear range, accuracy, and precision of the target compounds. In addition, UHPLC was used for chromatographic separation of 20 PAHs and nine nitro-PAHs, with the advantage of a shorter analytical time and superior resolution and sensitivity compared to the traditional HPLC^29^. Finally and most importantly, this is the first manuscript presenting UHPLC coupled with APPI-MS/MS for analysis of as many as 29 PAHs and nitro-PAHs.

## Experimental Section

### Choice of Target Compounds

The selected PAHs and nitro-PAHs are either source markers, abundant in the ambient air, or with toxicological significance. Besides, feasibility of LC-APPI-MS/MS analysis is considered. All priority PAHs were selected except naphthalene, due to its high vapor pressure. It was shown that GC-MS is a better analytical choice for naphthalene^19^,^30^,^31^. The 15 priority PAHs selected were acenaphthylene (ACPY), acenaphthene (ACP), fluorene (FLU), phenanthrene (PHEN), anthracene (ANTHR), fluoranthene (FL), pyrene (PYR), benz[*a*]anthracene (BAA), chrysene (CHRY), benzo[*b*]fluoranthene (BBF), benzo[*k*]fluoranthene (BKF), benzo[*a*]pyrene (BAP), dibenz[*a*,*h*]anthracene (DAA), benzo[*ghi*]perylene (BGHIP), and indeno[1,2,3-*cd*]pyrene (IND). In addition to 15 priority PAHs, five other PAHs were chosen for reasons given below. Benzo[*e*]pyrene (BEP) is used as a reference PAH to assess temporal variability and degradation patterns of other PAHs in environmental media due to its stability^3^; while cyclopenta[*cd*]pyrene (CPP) is classified as a probable human carcinogen^8^. Both were frequently analyzed in environmental studies. Moreover, coronene (COR) and benzo[*b*]naphtho[1,2*-d*]thiophene (BNT) were proposed as source markers for gasoline emissions and traffic emissions, respectively^32^,^33^,^34^. Lastly, retene (RET) is a known marker for biomass burning^33^,^35^. These markers are important in source apportionment studies^34^.

Moreover, nine nitro-PAHs were selected: 2 nitrofluorene (2NFLU), 2 nitrofluoranthene (2NFL), 3 nitrofluoranthene (3NFL), 1 nitropyrene (1NP), 2 nitropyrene (2NP), 4 nitropyrene (4NP), 6 nitrochrysene (6NCHRY), 7 nitrobezn[*a*]anthracene (7NBAA), and 6 nitrobenzo[*a*]pyrene (6NBAP). Among them, 2NFLU, 1NP, 4NP, and 6NCHRY are classified as possible human carcinogens^6^,^8^; they are the only mono-nitro-PAHs with such a high toxicity. The other five nitro-PAHs were selected because they were found in air and diesel particulate samples in substantial amounts^14^,^20^,^36^.

### Chemicals and Reagents

Certified reference material (CRM) was purchased from National Institute of Standards and Technology (SRM 1649b, Maryland, USA). This CRM is an atmospheric particulate material collected in an urban area containing both PAHs and nitro-PAHs. It was used to evaluate the applicability of this analytical method for real samples. Individual standards for 15 priority PAHs (99%), BEP and COR (50 μg/mL in toluene), and a PAH mixture (200 μg/mL in 1:1 dichloromethane/methanol) were purchased from AccuStandard (New Haven, CT, USA). Standards for BNT and CPP (10 μg/mL in cyclohexane), a five-PAH isotope mixture (NAP-*d*8, ACP-*d*10, PHEN-*d*10, CHRY-*d*12, and perylene-*d*12 (PERY-*d*12), 1000 ng/μL in toluene), BAA-*d*12 (10 ng/μL in acetonitrile), musk xylene-*d*15 (MX-*d*15, 100 ng/μL in acetone), 4NP (99.8%), 6NCHRY (99.4%), 7NBAA (10 μg/mL in cyclohexane), and 6NBAP (99.8%) were obtained from Dr. Ehrenstorfer GmbH (Augsburg, Germany). RET (99%) was purchased from City Chemical LLC (West Haven, CT, USA) and 1NP-*d*9 (99.2%) from C/D/N Isotopes Inc. (Quebec, Canada). 2NFLU (98%), 1NP (99%), and 2,4-difluoroanisole (98%) were purchased from Sigma-Aldrich (St. Louis, MO, USA). 2NFL (100 ng/mL), 3NFL (>97.5%), and 2NP (100 ng/mL) were obtained from Chiron (Trondheim, Norway). Acetonitrile (LC/MS grade, 0.2 μm prefiltered), methylene chloride (ultra resi-analyzed) and chlorobenzene (99.5%, analytical grade) were purchased from J. T. Baker (Phillipsburg, NJ, USA). Bromobenzene (99.5%) and anisole (99%) were obtained from Merck (Hohenbrunn, Germany) and Alfa Aesar (Lancashire, UK), respectively. Milli-Q water (Millipore, Tokyo, Japan) was used for UHPLC analysis.

### Instrument Settings and Dopant Choice

The mass spectrometer was an API 3000 triple quadrupole from Applied Biosystems/MDS SCIEX, equipped with a PhotoSprayTM APPI source (Toronto, Canada). The APPI source’s transfer voltage was 1500 V. Its probe temperature was 400 °C and the flow rate of the lamp gas was 1.5 L/min. Nitrogen was used for the nebuliser, drying, curtain, and collision gases. The settings for the nebuliser, curtain and collision gases were 6, 8, and 7 (instrument units), respectively. A Waters UHPLC system (Acquity UPLC, Waters Corporation, Milford, MA, USA) was used for LC analysis. Control of the instruments, data acquisition, and analysis were performed with Analyst software version 1.4.2.

The MS/MS parameters were optimized by manual tuning to obtain the best response signals via ramping various electric potentials. Standard solutions with 0.5 μg/mL concentrations in acetonitrile were infused into the MS at 20 μL/min. The scan type was “Multiple Reaction Monitoring” (MRM). The polarity was positive for 20 PAHs and six nitro-PAHs and negative for the other three nitro-PAHs (Table 1). The Q1 and Q3 resolutions were “unit” (0.7 ± 0.1 amu). The dwell time for each mass was 50 ms. For the infusion experiments, all scan parameters were the same except that the dwell time was 200 ms.

The choice of dopant is critical for effective ionization. Toluene, anisole, chlorobenzene, bromobenzene, and 2,4-difluoroanisole (DFA) were potential effective dopants for analysis of nonpolar compounds under reversed-phase LC conditions^21^,^37^,^38^. Thus, these were tested as dopants in analysis. Test results are presented for three dopant solutions: 0.5% anisole in toluene (dopant A), 0.5% DFA in bromobenzene (dopant B), and 0.5% DFA in chlorobenzene (dopant C).

### Column Separations and Analysis

For determination of PAHs and nitro-PAHs, good chromatographic separation is essential to differentiate isomeric compounds in the MS owing to their nearly identical fragmentation. Different analytical columns and separation conditions were investigated with the aim of achieving a short separation time and high selectivity and sensitivity. Sufficient separation of the target analytes was finally achieved with the conditions presented below.

Chromatographic separation was performed using a Pinnacle DB PAH 100 mm × 2.1 mm × 1.9 μm UHPLC column (Restek, Bellefonte, PA, USA) connected to an Acquity UPLC BEH C18 VanGuard Pre-column (2.1 mm × 1.7 μm, Waters). The column oven was maintained at 30 °C. The mobile phase solvents were 100% water (A) and 100% acetonitrile (B) with a flow rate of 300 μL/min. Dopant was delivered at one tenth of that flow rate. The elution gradient was 50%(A)/50%(B) initially, 100%(B) at 8–15 minutes, and 50%(A)/50%(B) at 15.1–19 minutes in the positive mode with curve 6; while in the negative mode, the elution gradient was 50%(A)/50%(B) initially, 40%(A)/60%(B) at 5 minute, 30%(A)/70%(B) at 10 minute, 100%(B) at 12–15 minutes, and 50%(A)/50%(B) at 15.1–19 minutes with curve 6 except at 10–15 minutes with curve 9. Sample injection volume was 5 μL. An amount of 5 μL of standard solutions was injected on column using a 10 μL loop with the “partial loop with needle overfill” method. LOD is defined as the level with “signal to noise” ratio equal to 3; and the “signal to noise” ratio is calculated based on peak height to peak height comparison.

### Field Sampling and Extraction

Aerosol samples were collected at two locations to assess the applicability of this analytical method, National Taiwan University (NTU) in the center of Taipei city and Hua-Lin (HL) station in the downwind mountainous area with an elevation of 400 m. Samples for particulate matters with aerodynamic diameter equal to or less than 2.5 μm (PM_2.5_) were collected in daytime (8 am-8 pm) and nighttime (8 pm-8 am) on July 4–11 and August 15–21, 2011. At each location, a high-volume sampler with single stage cascade for PM_2.5_ (Tisch Environmental Inc., Cleves, OH, USA) was used at a flowrate of 1.13 m^3^/min with quartz filters (8 in×10 in, Pall Life Sciences, Ann Arbor MI, USA) pre-baked at 900 °C overnight.

After sampling, one-fourth of the filter samples was spiked with 20 ng of the surrogate standards (the five-PAH isotope standards and 1NP-*d*9) and ultrasonically extracted with 20 mL of hexane/methylene chloride (1:4) for 30 minutes three times. The extracts were combined, concentrated to 0.5 mL with nitrogen, and purified using Waters HLB cartridges (6 mL volume, 500 mg bed mass) that were pre-conditioned with 6 mL each of methanol, methylene chloride, and hexane. The purified extracts were then filtered with 0.22 μm porosity PTFE filters (Great Engineering Technology Corp., Taipei, Taiwan), solvent-exchanged to 0.2 mL acetonitrile, spiked with 10 ng of the internal standards (BAA-*d*12 and MX-*d*15), and analyzed with the presented UHPLC-APPI/MS/MS method. A seven-point calibration curve was prepared in acetonitrile. Seven matrix spikes were prepared; each with one-fourth of the filter samples which were spiked with 20 ng of the target analytes and the surrogate standards. Three laboratory blanks were prepared by spiking 20 ng of the surrogate standards in one-fourth of the pre-baked filters. Matrix spikes and laboratory blanks went through the same analytical procedures as the filter samples. Two batches of CRM were extracted and analyzed in the same way; one batch (n = 7) for PAHs and another batch (n = 7) for nitro-PAHs. For PAHs, 20 mg of CRM was placed in a glass vial, spiked with 20 μL (1 ng/μL) of the surrogate standards, mixed by a Vortex mixer (Digisystem laboratory instrutments INC, Taiwan) for 30 seconds, then went through the same pre-treatment and analysis procedures as stated above. For nitro-PAHs, 200 mg of CRM were used following the same procedures.

## Results and Discussion

### Identification and Separation

The MS/MS parameters were optimized and two pairs of precursor/product ions of these compounds were obtained (Table 1), except the second product ions of COR, 2NFL, and 3NFL. The signals of the previously-reported second product ions of six PAHs (BAA, BBF, BKF, BAP, IND, DAA) using Acquity tandem MS from Waters Corporation were not strong enough for confirmation in API 3000^22^. Thus, different second product ions are offered. Moreover, for the 15 USEPA priority PAHs and 1NP, the second product ions were also reported in earlier studies 11,23,25,26,27, Table 1 provides an alternative set of product ions using a widely used instrument. Furthermore, for BEP, CPP, RET, BNT, and six nitro-PAHs, the second pairs of precursor/product ions in LC-MS/MS are reported for the first time; they are essential for confirmation.

**Figure 1:**
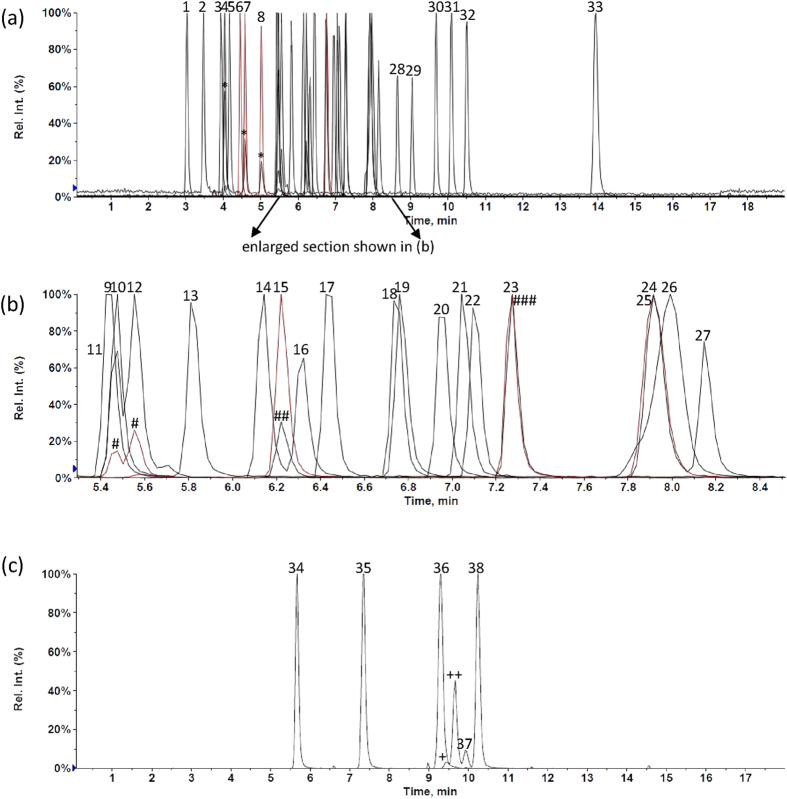
MRM ion chromatograms of target compounds and deuterated standards with on-column injection amounts of 300pg each; retention time (RT) for positive mode (**a,b**): (1) 3.03, NAP-*d*8; (2) 3.47, ACPY; (3) 3.94, ACPY-*d*10; (4) 4.04, ACP; (5) 4.17, FLU; (6) 4.45, PHEN-*d*10; (7) 4.58, PHEN; (8) 5.02, ANTHR; (9) 5.44, 1NP-*d*9; (10) 5.47, FL; (11) 5.47, 4NP; (12) 5.55, 1NP; (13) 5.82, PYR; (14) 6.14, 7NBAA; (15) 6.22, 2NP; (16) 6.32, 6NCHRY; (17) 6.44, BNT; (18) 6.77, CPP; (19) 6.75, BAA-*d*12; (20) 6.95, BAA; (21) 7.05, CHRY-*d*12; (22) 7.27, CHRY; (23) 7.27, RET; (24) 7.92, PERY-*d*12; (25) 7.92, BEP; (26) 7.97, 6NBAP; (27) 8.15, BBF; (28) 8.65, BKF; (29) 9.05, BAP; (30) 9.68, DAA; (31) 10.9, BGHIP; (32) 10.5, IND; (33) 13.94, COR; RT for negative mode (c): (34) 5.67, 2NFLU; (35) 7.35, MX-*d*15; (36) 9.30, 1NP-*d*9; (37) 9.93, 2NFL; (38) 10.24, 3NFL; *other MRM (m/z:152.2–>126.0) for ACP, PHEN and ANTHR, #other MRM (247.2–>201.1) for 4NP and 1NP; ##other MRM (247.2–>189.1) for 2NP; ###benzo[*b*]naphtho[2,3-*d*]thiophene, not a target analyte; +: 4NP and ++: 1NP in the negative mode.

Figure 1 shows MRM ion chromatograms of these target analytes and deuterated standards with on-column injection amounts of 300 pg each. The 29 PAHs and nitro-PAHs are well separated and quantified in 15 minutes in the positive mode and 11 minutes in the negative mode. Compared to 45 minutes required for separating these 29 target analytes in GC/MS (data not shown), this UHPLC-APPI-MS/MS method (totally 26 minutes) cut down the analysis time to one half.

If only 15 priority PAHs are analyzed, it takes only 5 minutes using a shorter column (Pinnacle DB PAH 50 mm × 2.1 mm × 1.9 μm) with 600 μL/min at 30 °C (data not shown); it is close to 3.5 minutes reported for 16 priority PAHs with Waters Acquity tandem MS^19^. However, in order to separate BEP from the other isomers (BBF, BKF, and BAP), 100 mm long column was used instead, resulting in a slower flow rate (300 μL/min) and longer analysis time. BEP, not a priority pollutant, is a relatively stable PAH. It is essential to separate and identify these four isomers in real samples since they are frequently found in the air and their carcinogenic potentials differ significantly. BBF and BKF exhibit only 6–14% and 3–10% of BAP’s (a human carcinogen^4^) cancer-causing potential, respectively; while BEP exhibits very little toxicity^3^. Thus, our method provides accurate concentrations for these four isomers compared to other methods concerning only priority PAHs, which may overestimate the actual concentrations of more-toxic PAHs. As a result, the subsequent health risk assessment will be overestimated as well.

### Limits of Detection with Different Dopants

Table 2 and 3 shows the LODs of 29 target compounds with three different dopant solutions. In general, the best sensitivity is associated with dopant C (0.5% DFA in chlorobenzene). All the LODs of PAHs are below 10 pg except ACPY; all the LODs of nitro-PAHs are below 3 pg except 2NFL. Compared to those with dopants A and B, the LODs with dopant C are much better for ACPY and FLU and are comparable for other PAHs and nitro-PAHs. Further evaluation of linearity, accuracy, and precision were conducted with dopants A and C only.

In comparison, the LODs of PAHs with dopant C (with the best sensitivity) are all lower than those using GC-EI-MS/MS in the literatures^31^, except ACPY, the one with the highest vapor pressure among the analyzed PAHs (Table 2). Especially for some high-m.w. PAHs, our LODs are one order of magnitude lower. Compared to LODs using other LC-MS and LC-MS/MS systems with other dopants 11,19,22,23,24,25,26,28,30,39, the LODs with dopant C are either lower by one order of magnitude or at least comparable. The results demonstrate the advantage and applicability of our analytical method, with LODs lower than or comparable to those previously reported using GC-MS/MS, LC-MS, and LC-MS/MS systems. Furthermore, this work presents LODs of 4 important PAH compounds (BNT, CPP, RET, and COR) which have never been analyzed by LC-APPI-MS/MS.

Moreover, the LODs of nitro-PAHs with dopant C, in the range of 0.3–2.8 pg with one exception of 11.3 pg, are lower than or comparable to those using other LC-related and GC-related methods but higher than those using GC-NICI-MS in the literatures (Table 3) 14,15,40,41,42. Typically, the lowest concentrations of these individual nitro-PAHs in ambient air are around 0.4–1.0 pg/m^3^ 8,14,20. With a typical 24-hr sampling of 1400 m^3^ of air and final sample volume of 200 μL with 5 μL injection, the final injection amounts are in the range of 14–35 pg. Therefore, our LODs are low enough for nitro-PAH quantification in ambient air samples. Furthermore, it took 25 minutes using GC-NICI-MS to analyze 11 nitro-PAHs including four analytes here^15^; the last eluted nitro-PAH in their method was 6NCHRY, which eluted in 6.3 minutes using our method. The relatively shorter analytical time makes this presented method an excellent alternative for nitro-PAHs. More importantly, this method analyzes nitro-PAHs simultaneously with PAHs.

### Linearity, Accuracy, and Precision

The linear ranges of responses were assessed from levels close to LODs to 200 or 500 ng/mL, covering 2 orders of magnitude for most species (Table 4). The R^2^ of these linear calibration curves were all greater than 0.995. Moreover, the accuracy and precision were evaluated with repeated injections of standard solutions prepared at two different concentrations (10 ng/mL and 200 ng/mL for most species, Table 4). The accuracy and precision with both dopants A and C are within 8% variability for both low and high concentrations, with few exceptions. In summary, the results show the PAH and nitro-PAH responses were linear over two orders of magnitude with fairly good precision and accuracy with both dopants A and C.

### Certified Reference Materials

Based on the above assessment, the analytical method with dopant C has the best sensitivity and a two-order-of-magnitude linear range with good accuracy and precision. Thus, CRM in replicates (n = 7) were analyzed with this method. The obtained concentrations of 20 PAH and nine nitro-PAH species are compared with NIST certified values (Table 5). The percent difference between the analyzed concentrations and the certified values are all less than 10.7%. The standard deviations (SD) of all analyzed species are mostly comparable with those NIST values. These results show that the combination of the presented extraction and analytical method provides sensitive, specific, and reliable results. Thus, this presented method is suitable for PAHs and nitro-PAHs analysis of urban aerosols.

### Field Sample Evaluation

Fifty-eight successful PM_2.5_ filter samples were collected at NTU and HL sites; they were extracted and analyzed with the same method as CRM. The concentrations of total PAHs (sum of the analyzed 20 PAH species) were 827.8 ± 255.9 and 343.0 ± 167.0 pg/m^3^ in the daytime at NTU and HL, respectively; the corresponding nighttime levels were 1393.9 ± 852.7 and 334.0 ± 177.1 pg/m^3^. The urban site (NTU) had concentrations 2.4–4.2 times of those in the downwind mountainous site (HL). For the total nitro-PAHs (sum of the nine analyzed nitro-PAHs), the concentrations in the daytime at NTU and HL were 4.3 ± 2.9 and 4.2 ± 3.1 pg/m^3^, respectively; the corresponding levels in the night time were 9.1 ± 4.3 and 5.7 ± 2.0 pg/m^3^. The total nitro-PAHs at urban site (NTU) in the daytime were in the same ranges as those in the downwind mountainous site (HL); while those levels in the night time were 1.6 times higher than those at HL. In addition, the concentrations of both total PAHs and total nitro-PAHs were higher in the night time compared to those in daytime at both locations, possibly due to the lower boundary layer height in the night time.

For individual species, the detectable percentages (%) are listed in Table 5. Most of the PAHs were 100% above the LODs in the real samples. The % above the LODs was 0 for ACPY, ACP, FLU, 2NFLU, 2NFL, 3NFL, and 6NCHRY. ACPY, ACP, and FLU may be predominantly present in the gaseous phase rather than the aerosol phase in summer time due to their high vapor pressures^1^. The levels of nitro-PAHs in the air are usually lower than those of PAHs with lower % of detectable^36^; our results were consistent with the previous findings.

**Figure 2:**
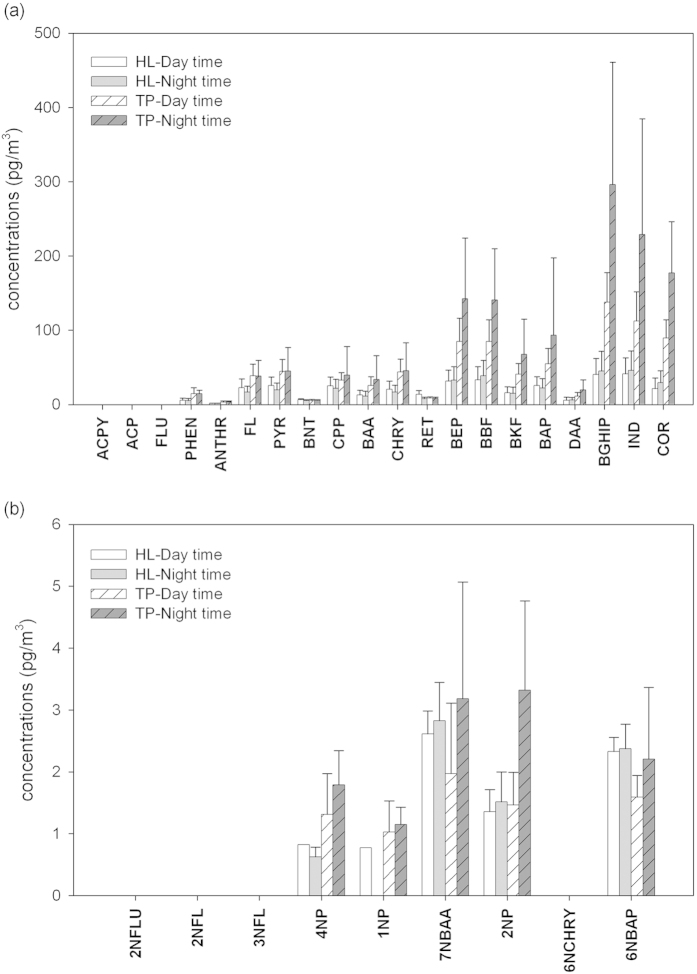
Analyzed results for PM_2.5_ samples at NTU (urban) and HL (downwind mountain) sites for (**a**) PAHs and (**b**) nitro-PAHs.

The concentrations of individual PAH and nitro-PAH species are shown in Fig. 2; the concentrations of non-detectable were treated as zero. The levels of individual PAH species in the aerosol phase were 0.28–785.2 pg/m^3^. The reported PAH range in ambient air in Taipei was 10–420 pg/m^3^ 43; the highest level obtained in this work is in the same order of magnitude while the lowest level is two orders of magnitude lower, possibly due to our lower LODs than the previous work. The levels of nitro-PAHs in the aerosol phase in this work were 0.19–6.8 pg/m^3^. The reported levels of 1NP, 2NP, and 7NBAA were 1.4–127, 0.8–70, and 0.7–33 pg/m^3^, respectively^20^,^36^; the lowest levels in this work are in the same order of magnitude as theirs and the highest levels are one order of magnitude lower than theirs. The blank values were below the LODs except for PHEN (1.1 ± 0.7 pg/m^3^, n = 3). The recovery rates of the spiked surrogates were 85–106%. The presented results were corrected by blank values and recovery rates. The mean recovery rates of these target compounds in matrix spikes (n = 7) were 80.5–125%. No strong matrix effect was observed. The filter samples (n = 6) were also analyzed in duplicates; the precision of duplicates was mostly within 10%. In summary, the above results indicate that this analytical method is well applicable in environmental studies.

Figure 2 clearly shows that the concentrations of BEP are in the same range as those of BAP. It is essential to separate and identify both BEP and BAP in the real samples in order to accurately conduct health risk assessment based on the measured concentrations since their toxicities differ substantially. With analytical methods only focusing on the EPA priority PAHs, BEP may be mistakenly taken as BAP since they are isomers with the same pairs of precursor/product ions. The estimated health risks may be overestimated by two-folds as indicated in this work. With the presented analytical methods, accurate BEP and BAP levels are assessed; health risk assessment can be conducted and the effective control strategy can be formulated accordingly.

## Conclusions

The presented method is the first UHPLC-APPI-MS/MS method capable of simultaneously analyzing 29 environmentally and toxicologically important PAH and nitro-PAH species in aerosols. With a Pinnacle DB PAH 100 mm × 2.1 mm × 1.9 μm UHPLC column and a water/acetonitrile binary mobile phase, the 29 target analytes are separated in 15 minutes in the positive mode and 11 minutes in the negative mode, one half of GC/MS analysis time. In addition, the second pairs of precursor/product ions in LC-MS/MS are reported for these compounds, which is essential for confirmation. For ten compounds, these are reported for the first time. This method separates and quantifies four isomers (BBF, BKF, BAP, and a non-priority BEP) to avoid overestimation of toxin levels; this demonstrates its importance for health-related researches. Furthermore, the best sensitivity is associated with 0.5% DFA in chlorobenzene as the dopant; all LODs of PAHs are below 10 pg except ACPY; all the LODs of nitro-PAHs are below 3 pg except 2NFL. The responses were linear over two orders of magnitude with fairly good accuracy and precision. Certified reference materials and real samples were analyzed to demonstrate its applicability. In summary, a fast, sensitive, and reliable UHPLC-APPI-MS/MS method is presented for 29 environmentally and toxicologically important PAHs and nitro-PAHs, expanding the analysis scope beyond 16 priority PAHs. This method has wide application in health-related air pollution studies.

## Additional Information

**How to cite this article**: Lung, S.-C. C. and Liu, C.-H. Fast analysis of 29 polycyclic aromatic hydrocarbons (PAHs) and nitro-PAHs with ultra-high performance liquid chromatography-atmospheric pressure photoionization-tandem mass spectrometry. *Sci. Rep.* **5**, 12992; doi: 10.1038/srep12992 (2015).

## Figures and Tables

**Table 1:** Two pairs of precursor/product ions and the optimized MS/MS parameters for (a) PAH and (b) nitro-PAHs species.

(a)										
Compound (abbreviation)	Q1	Q3	**MRM1**					**MRM2**		
			**DP**	**FP**	**EP**	**CE**	**CXP**	**2^nd^ Q3**	**CE**	**CXP**
			**(Volts)**						**(Volts)**		
NAP-*d*8	136.3	108.1	30.8	120.0	11.6	42.9	8.8	134.2	40.7	8.0
ACPY	152.2	126.0	116.8	134.0	10.6	45.8	6.1	150.0	49.1	7.1
ACP-*d*10	162.2	160.1	81.5	159.0	10.3	36.8	7.8	158.1	38.0	7.1
ACP	154.2	153.1	42.8	193.8	10.0	31.8	11.0	152.1	45.1	8.0
FLU	165.2	115.1	70.3	351.9	11.0	45.5	9.1	139.1	46.9	6.2
PHEN-*d*10	188.3	160.1	29.1	161.1	5.2	46.0	7.2	186.2	41.1	9.1
PHEN	178.2	176.1	15.5	83.0	5.1	49.9	8.3	152.0	48.1	7.0
ANTHR	178.2	176.1	15.5	83.0	5.1	49.9	8.3	152.0	48.1	7.0
FL	202.2	200.0	22.0	93.8	5.0	63.5	10.1	176.1	58.9	12.9
PYR	202.2	200.0	22.0	93.8	5.0	63.5	10.1	176.1	58.9	12.9
BNT	234.3	232.2	8.1	59.8	10.0	56.3	12.0	202.1^a^,^c^	46.3	10.8
CPP	226.3	224.1	14.9	59.8	10.9	68.0	10.0	200.1^c^	64.3	10.0
BAA-*d*12	240.3	236.2	39.9	189.0	5.2	56.4	14.0	238.2	47.2	14.0
BAA	228.3	226.1	25.3	114.9	10.6	55.6	15.8	202.1	52.0	15.0
CHRY-*d*12	240.3	236.2	39.3	189.0	5.2	56.4	14.0	238.3	47.2	14.0
CHRY	228.3	226.1	25.3	114.9	10.6	55.6	15.8	202.1	52.0	15.0
RET	234.2	219.2	18.1	105.1	5.1	23.0	10.0	204.2^c^	25.0	10.9
PERY-*d*12	264.3	260.1	30.1	138.0	10.5	71.6	6.0	262.2	55.4	24.0
BEP	252.3	250.1	26.3	124.5	10.8	60.7	13.0	226.1^c^	59.1	10.9
BBF	252.3	250.1	26.3	124.5	10.8	60.7	13.0	226.1	59.1	10.9
BKF	252.3	250.1	26.3	124.5	10.8	60.7	13.0	226.1	59.1	10.9
BAP	252.3	250.1	26.3	124.5	10.8	60.7	13.0	226.1	59.1	10.9
DAA	278.3	276.1	25.0	89.9	10.6	61.9	14.1	250.1	81.8	18.9
BGHIP	276.3	274.1	17.3	80.3	10.8	71.8	14.7	250.1	66.4	13.0
IND	276.3	274.1	17.3	80.3	10.8	71.8	14.7	250.1	66.4	13.0
COR	300.3	298.2	25.0	107.0	10.2	80.4	12.9	—b	—	—
**(b)**										
Compound	Q1	Q3	**MRM1**					**MRM2**		
			**DP**	**FP**	**EP**	**CE**	**CXP**	**2^nd^ Q3**	**CE**	**CXP**
			**(Volts)**						**(Volts)**		
Positive mode											
1-nitropyrene (1NP)-*d*9	256.2	226.1	32.3	156.1	4.8	27.9	12.1	240.1	41.7	14.8	
4-nitropyrene (4NP)	247.2	189.1	23.4	118.7	7.7	43.1	8.7	201.1^c^	31.2	10.7	
1NP	247.2	189.1	23.4	118.7	7.7	43.1	8.7	201.1	31.2	10.7	
7-nitrobenzo[*a*]anthracene (7NBAA)	273.3	215.1	16.2	98.0	8.8	30.8	11.2	217.1^c^	23.0	11.5	
2-nitropyrene (2NP)	247.2	201.1	21.6	110.3	9.9	31.2	10.7	189.1^c^	43.1	8.7	
6-nitrochrysene (6NCHRY)	273.3	215.1	16.2	98.0	8.8	30.8	11.2	217.1^c^	23.0	11.5	
6-nitrobenzo[*a*]pyrene (6NBAP)	297.3	267.1	19.9	92.1	9.8	25.3	14.7	241.0^c^	28.4	12.4	
Negative mode											
2-nitrofluorene (2NFLU)	210.2	180.0	−23.4	−100.0	−6.3	−26.8	−9.5	164.0^c^	−31.0	−8.3	
MX^d^-*d*15	282.4	186.1	−12.3	−70.3	−8.0	−35.1	−10.4	234.2	−30.1	−12.6	
1NP-*d*9	256.3	226.3	−24.3	−119.9	−5	−18.4	−37.2	—	—	—	
2-nitrofluoranthene (2NFL)	247.3	217.0	−18.0	−100.7	−9.5	−20.7	−12.8	—	—	—	
3-nitrofluoranthene (3NFL)	247.3	217.0	−18.0	−100.7	−9.5	−20.7	−12.8	—	—	—	

Note: DP: declustering potential, FP: focusing potential, EP: entrance potential, CE: collision energy, and CXP: collision cell exit potential.

^a^for BNT, second pair of Q1 (219.3) and Q3 (202.1) were identified instead of second Q3 of the Q1 (234.3), the associated DP, FP, and EP are 72.1, 130.0, and 4.7, respectively.

^b^not found.

^c^reported for the first time.

^d^MX: musk xylene (1-tert-butyl-3,5-dimethyl-2,4,6-trinitrobenzene).

**Table 2:** LODs (signal to noise ratio (S/N) equal to 3, n = 3) of 20 PAHs using UHPLC-APPI-MS/MS in this work compared to those presented in previous publications with different dopant solutions and different instrumentation; dopant A: 0.5% anisole in toluene, dopant B: 0.5% DFA in bromobenzene, and dopant C: 0.5% DFA in chlorobenzene; values shown are injection amounts (pg).

**Ion source and MS**	**UHPLC-APPI-MS/MS (this work)**			**GC-EI-MS/MS [31]**	**LC-APPI-MS/MS [11]**^a^	**Capillary LC-APPI-MS/MS [26]**^b^	**HPLC-APPI-MS [30]**^c^	**HPLC-APPI-MS [39]**^c^	**HPLC-APPI-MS/MS [22]**	**HPLC-APPI-MS/MS [23]**	**HPLC-APPI-MS/MS [24]**	**HPLC-APPI-MS/MS [25]**^d^	**UHPLC-APPI-MS [28]**^e^	**UHPLC-APPI-MS/MS [19]**
**APPI Dopant**	**dopant A**	**dopant B**	**dopant C**		**Toluene and Anisole**	**Toluene**	**dopant A**	**Toluene**	**Toluene**	**Toluene**	**Toluene**	**Chloro-benzene**	**Chloro-benzene**	**Chloro-benzene**
ACPY	225	94	24	11	— ^f^	—	33	—	—	—	—	160	105.6	158.4
ACP	9.1	5.8	7.5	13	—	—	19	—	—	—	—	150	60.4	91.6
FLU	22	20	7.9	16	—	—	7.9	—	—	—	—	79	11.7	6.2
PHEN	7.7	7.9	4.4	9	—	—	12	15	—	—	461	190	10.2	4.3
ANTHR	5.7	8.7	6	10	—	5	11	13	—	—	768	290	10.4	3.1
FL	4.8	8.8	5.6	8	—	—	8.7	41	—	—	—	120	9.3	3.4
PYR	6.8	8.7	5.5	8	—	—	10	7.9	—	—	—	170	8	10.3
BNT	3.4	5	4.3	—	—	—	—	—	—	—	—	—	—	—
CPP	0.7	1.6	0.6	—	7.3	—	—	—	—	—	—	—	—	—
BAA	1.4	4.4	2	10	5	10	8.7	4	166	8.9	319	120	14.8	5.4
CHRY	1.3	3.6	0.9	8	8.4	—	9.4	4.1	—	—	—	110	13.9	11.4
RET	3.3	14	7.7	—	—	—	—	—	—	—	—	—	—	—
BEP	1.7	1.9	2.6	—	—	—	—	10	—	—	—	160	—	—
BBF	0.9	1.6	3	—	9	—	8.7	46	89	6.3	—	75	12.9	2.5
BKF	0.8	1.8	2.7	9	8.4	—	8.1	16	34	11	—	210	11.9	2.5
BAP	1	2.3	2.9	11	6.3	30	8.6	10	67	2.5	—	230	12.1	3.3
DAA	0.8	0.8	0.7	39	9	—	9.3	—	22	—	—	160	10.3	1.7
BGHIP	1.3	1.3	1.6	27	9.5	—	14	—	—	—	—	190	14.1	2.8
IND	1.3	1.2	3.2	28	7.1	—	13	7.5	—	—	—	260	11.2	4.4
COR	2.7	1.7	3	—	—	—	—	—	—	—	—	—	—	—

^a^LOD (pg) was converted based on the conversion factor listed in reference.

^b^LOD (pg) was converted from the unit of ng/mL in the references with 1 μl injection.

^c^LOD (pg) was converted from the unit of ng/mL in the references with 10 μl injection.

^d^converted from MDL with sample loop of 10 ml.

^e^sample matrix is oyster.

^f^not found.

**Table 3:** LODs (signal to noise ratio (S/N) equal to 3, n = 3) of nine nitro-PAHs using UHPLC-APPI-MS/MS in this work compared to those presented in previous publications with different dopant solutions and different instrumentation; dopant A: 0.5% anisole in toluene, dopant B: 0.5% DFA in bromobenzene, and dopant C: 0.5% DFA in chlorobenzene; values shown are injection amounts (pg).

**Ion source and MS**	**UHPLC-APPI-MS/MS**			**GC-NICI-MS [40]**	**GC-NICI-EC-MC/MS [41]**^c^	**LC-NICI-MS [42]**	**HPLC-PBI-MS [42]**	**UHPLC-APCI-TOF [15]**	**HPLC-APPI-MS/MS [23]**
**APPI Dopant**	**Dopant A**	**Dopant B**	**Dopant C**						**Toluene**
2NFLU	0.4	8.6	0.3	0.15	0.1–0.5	—b	70	5–115	—
2NFL	14.3	243	11.3	0.27	—	—	—	—	—
3NFL	1.2	65	1.1	0.16	0.1–0.5	—	—	5–115	—
4NP	1.7	1.7	1.2	—	—	—	—	5–115	—
1NP	1.6	0.7	0.8	0.17	0.1–0.5	5	1	5–115	4.8
7NBAA	2.4	3.2	2.8	0.3	—	—	—	5–115	—
2NP	0.5	0.3	0.5	1.8	—	—	—	5–115	—
6NCHRY	3	0.7	2	0.09	0.1–0.5	—	—	5-115	—
6NBAP	1.7	1.5	0.8	0.65	—	—	—	5-115	—

Note:

^a^LOD (pg) was converted from the unit of ng/mL in the references with 10 μl injection

^b^not found

^c^LOD ranges presented in the references

**Table 4:** The linear range, R^2^, accuracy and precision of (a) 20 PAHs and (b) nine nitro-PAHs using UHPLC-APPI-MS/MS with two different dopant solutions; accuracy and precision with dopants (n = 7) are presented at two concentration levels; accuracy: percentages of the obtained concentrations over the expected concentrations; precision: values in parenthesis are percent coefficient of variation (%CV) of the repeated injections.

Compound	**0.5% anisole in toluene (dopant A)**				**0.5% DFA in chlorobenzene (dopant C)**			
	**Linear Range (ng/mL)**	**R^2^**	**Accuracy and precision**		**Linear Range (ng/mL)**	**R^2^**	**Accuracy and precision**	
			**10 ng/mL**	**200 ng/mL**			**10 ng/mL**	**200 ng/mL**
ACPY	50–500	0.9978	98.8^a^ (7.6 )	96.1(6.1)	20–500	0.9966	96.7^b^ (8.8)	94.1(4.3)
ACP	5–200	0.9998	98.0(3.6)	103.2(2.9)	5–200	0.9996	94.6(7.8)	94.8(4.1)
FLU	20–500	0.9954	103^b^ (2.5 )	100.8(4.6)	10–500	0.9968	101(9.5)	95.4(5.4)
PHEN	2–500	0.997	101(6.0)	97.1(3.8)	5–500	0.9988	102(9.0)	94.6(4.7)
ANTHR	2–500	0.9976	101(5.1)	99.4(3.3)	5–500	0.998	96.8(5.1)	93.7(3.3)
FL	2–200	0.9978	107(4.1)	103.2(3.1)	5–500	0.9958	94.5(6.6)	98.8(2.8)
PYR	2–200	0.9994	105(3.8)	103.8(2.3)	5–500	0.9964	95.0(5.5)	94.4(3.7)
BNT	2–500	0.9982	98.2(7.4)	107.4(2.7)	5–100	0.9992	108(5.9)	103^3^ (7.8 )
CPP	2–500	0.9996	98.6(6.5)	105(3.8)	2–200	0.9998	93.1(5.8)	97.5(2.2)
BAA	2–500	0.9996	99.9(7.6)	104.1(3)	2–200	0.9984	98.4(5.8)	102(3.9)
CHRY	2–500	0.9994	98.9(5.3)	103.9(2.5)	2–200	0.9994	97.4(7.3)	102(4.6)
RET	2–200	0.9974	94.9(5.4)	99.8(3.8)	10–500	0.9978	93.5(8.3)	94.9(2.7)
BEP	2–500	0.9968	95.2(4.5)	106(2.6)	2–200	0.9998	99.9(5.7)	104(4.5)
BBF	2–500	0.9958	100(6.8)	100.5(1.8)	2–200	0.9996	105(6.2)	103(4.5)
BKF	2–500	0.9986	96.9(5.6)	104.8(3.1)	2–200	0.9992	92.0(5.2)	104(4)
BAP	2–500	0.998	98.4(3.8)	97.7(4.2)	2–200	1.00	98.4(7.2)	93.8(6.2)
DAA	2–500	0.9972	106(5.0)	102.2(2.2)	2–200	0.998	94.7(3.8)	94.6(3.9)
BGHIP	2–500	0.9988	101(3.9)	100.4(2.5)	2–200	0.998	94.1(3.6)	93.1(6.4)
IND	2–500	0.9994	99.1(4.9)	103.4(3.2)	2–200	0.9998	92.6(8.9)	97.8(6.2)
COR	2–500	0.9974	98.9(4.4)	107.5(3.4)	2–200	0.9998	92.1(4.1)	103(6.7)
								
(b)								
2NFLU	1–200	0.9988	98.8(3.7)	97.8(2.1)	1–200	0.9998	98.5(1.9)	104(2.5)
2NFL	5–200	0.9974	102(7.6)	99.4(4.1)	5–200	0.9994	99.9(6.6)	99.4(1.9)
3NFL	1–200	0.9985	99.1(3.4)	96.4(4.4)	1–200	1.00	97.2(3.9)	106(5.5)
4NP	1–200	0.9988	109(5.4)	98.2(3.5)	1–200	0.9952	94.6(3.7)	104(7.5)
1NP	1–200	0.995	102(6.5)	102.0(5.5)	1–200	0.9996	96.0(2.3)	102(6.7)
7NBAA	1–200	0.9992	92.1(3.4)	104.0(2.7)	1-200	0.997	96.2(8.2)	98.2(3.0)
2NP	1–200	0.998	97.6(4.5)	99.5(3.1)	1-200	0.9984	94.9(6.6)	96.1(4.2)
6NCHRY	1–200	0.9994	95.6(5.7)	99.1(4.7)	1-200	0.9994	95.4(6.0)	94.0(3.9)

^a^50 ng/mL.

^b^20 ng/ml.

^c^100 ng/ml.

**Table 5:** The analyzed results of NIST 1649B (n = 7) and the percentages of detectable (above the LODs) PAHs and nitro-PAHs levels in real samples (n = 58).

**Compound**	**analyzed**	**certified**	% difference	% detectable in real samples
**(a)**	**Mean (SD) (mg/kg)**	**Mean (SD) (mg/kg)**		
ACPY	0.198(0.029)	0.184(0.026)	7.6	0%
ACP	0.197(0.027)	0.192(0.036)	2.4	0%
FLU	0.209(0.036)	0.222(0.016)	5.8	0%
PHEN	3.91(0.329)	3.94(0.047)	0.78	100%
ANTHR	0.41(0.059)	0.403(0.002)	1.8	74%
FL	6.14(0.858)	6.14(0.12)	0.02	100%
PYR	4.76(0.39)	4.78(0.029)	0.53	100%
BNT	−a	−a	−a	26%
CPP	0.255(0.027)	0.235(0.06)	8.4	100%
BAA	2.04(0.192)	2.09(0.048)	2.5	100%
CHRY	2.96(0.28)	3.01(0.044)	1.5	100%
RET	0.204(0.039)	0.226(0.014)	9.6	100%
BEP	2.94(0.207)	2.97(0.043)	1.1	100%
BBF	6.24(0.791)	5.99(0.2)	4.2	100%
BKF	1.8(0.091)	1.75(0.083)	3.2	100%
BAP	2.55(0.315)	2.47(0.17)	3.1	100%
DAA	0.259(0.036)	0.29(0.028)	10.7	83%
BGHIP	3.97(0.411)	3.94(0.052)	0.76	100%
IND	2.92(0.397)	2.96(0.017)	1.2	100%
COR	2.73(0.222)	2.83(0.46)	3.7	98%
(b)	(mg/kg)	(mg/kg)		
2NFLU	−a	−a	−a	0%
2NFL	308(15)	311(5)	1.1	0%
3NFL	4.4(0.3)	4.6(0.1)	5.4	0%
4NP	5.2(0.5)	5.5(0.1)	6.1	28%
1NP	71.1(2.9)	71.8(1.3)	0.98	19%
7NBAA	24(2.2)	24.2(0.7)	0.88	81%
2NP	11.5(0.6)	10.8(0.3)	6.7	90%
6NCHRY	4.1(0.6)	3.8(0.1)	7.3	0%
6NBAP	−a	−a	−a	59%

^a^not available.
